# Alpinetin: A Review of Its Pharmacology and Pharmacokinetics

**DOI:** 10.3389/fphar.2022.814370

**Published:** 2022-02-04

**Authors:** Ge Zhao, Yue Tong, Fei Luan, Wenjing Zhu, Chenglin Zhan, Tiantian Qin, Weixiao An, Nan Zeng

**Affiliations:** ^1^ State Key Laboratory of Southwestern Chinese Medicine Resources, School of Pharmacy, Chengdu University of Traditional Chinese Medicine, Chengdu, China; ^2^ Department of Pharmacy, Affiliated Hospital of Southwest Medical University, Luzhou, China; ^3^ Department of Pharmacy, Chengdu Second People’s Hospital, Chengdu, China

**Keywords:** alpinetin, flavonoids, mechanisms of action, pharmacokinetics, pharmacology

## Abstract

Flavonoids isolated from medicinal herbs have been utilized as valuable health-care agents due to their virous biological applications. Alpinetin is a natural flavonoid that emerges in many widely used medicinal plants, and has been frequently applied in Chinese patent drugs. Accumulated evidence has demonstrated that alpinetin possesses a broad range of pharmacological activities such as antitumor, antiinflammation, hepatoprotective, cardiovascular protective, lung protective, antibacterial, antiviral, neuroprotective, and other properties through regulating multiple signaling pathways with low systemic toxicity. However, pharmacokinetic studies have documented that alpinetin may have poor oral bioavailability correlated to its extensive glucuronidation. Currently, the reported pharmacological properties and pharmacokinetics profiles of alpinetin are rare to be scientifically reviewed. In this article, we aimed to highlight the mechanisms of action of alpinetin in various diseases to strongly support its curative potentials for prospective clinical applications. We also summarized the pharmacokinetics properties and proposed some viable strategies to convey an appreciable reference for future advances of alpinetin in drug development.

## Introduction

Flavonoids are a large number of polyphenolic substances including a fundamental configurational unit of 2-phenylchromone, that can be found in the plants’ flowers, leaves, stems or the fruits (Ji et al., 2020; Wen et al., 2021). Natural flavonoids are principally classified into chalcones, flavones, flavonols, flavanols, flavans, flavanones, anthocyanidins, isoflavonoids, and others on the basis of heterocyclic ring substituted patterns (Wen et al., 2017). To date, more than 4,000 varieties of identified flavonoids have been characterized (Kumar and Pandey, 2013). Simultaneously, flavonoid monomers have been emphasized and gained extensive attention attributed to their versatile biological applications and potential medicinal values, such as anticancer, antidiabetic, protective effects against mitochondriopathies and associated pathologies, antiviral, antibacterial, anti-inflammatory properties, protective effects against autoimmune and cardiovascular diseases, and anti-oxidant. (Pietta, 2000; AI-Ishaq et al., 2019; Rengasamy et al., 2019; Ciumărnean et al., 2020; Čulenová et al., 2020; Koklesova et al., 2021; Liskova et al., 2021).

**FIGURE 1:**
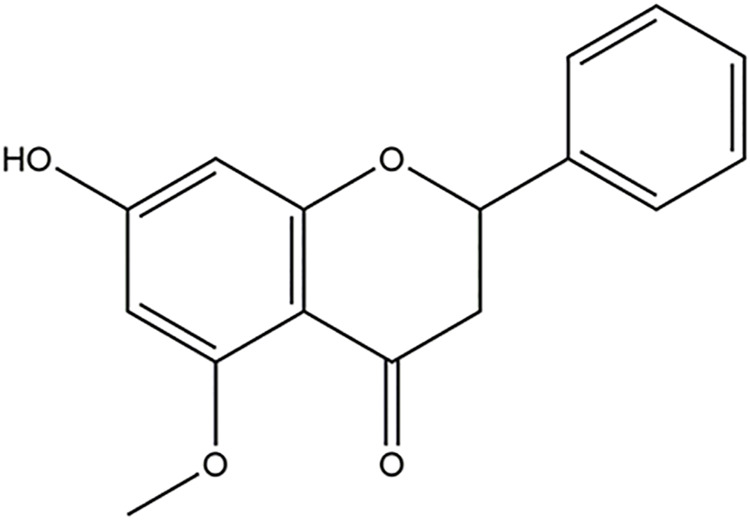
The chemical structure of alpinetin.

Alpinetin (7-hydroxy-5-methoxyflavanone; C_16_H_14_O_4_; Figure 1), a natural dihydroflavone, was firstly extracted from the plants of *Alpinia intermedia* Gagnep. [Zingiberaceae] in Japan (Qiao et al., 2001). For decades, alpinetin was also widely found in many other medicinal herbs and has been abstracted from *Alpinia hainanensis* K. Schum. [Zingiberaceae] (Rao and Lin, 1998; Liang et al., 2007; Liu et al., 2007; Wang et al., 2007; Liu and Wu, 2009; Li et al., 2010; Zou and Lu, 2011; Zhao et al., 2013; Bi et al., 2018), *Alpinia mutica* Roxb. [Zingiberaceae] (Malami et al., 2016; Malami et al., 2017a), *Alpinia pinnanensis* T. L. Wu & S. J. Chen [Zingiberaceae] (Giang et al., 2005), *Amomum subulatum* Roxb. [Zingiberaceae] (Rao et al., 1976; Singh et al., 2015), *Boesenbergia rotunda* (L.) Mansf. [Zingiberaceae] (Tewtrakul et al., 2003; Morikawa et al., 2008; Wangkangwan et al., 2009; Tan et al., 2015; Liew et al., 2020; Nguyen et al., 2020), *Campomanesia phaea* (O.Berg) Landrum [Myrtaceae] (Lorençoni et al., 2020), *Carya cathayensis* Sarg. [Juglandaceae] (Cao et al., 2012), *Combretum albopunctatum* Suess. [Combretaceae] (Katerere et al., 2004), *Dalbergia odorifera* T.C.Chen [Fabaceae] (Zhao et al., 2020), *Dalbergia parviflora* Roxb. [Fabaceae] (Umehara et al., 2008), *Mikania micrantha* Kunth [Asteraceae] (Jiang et al., 2001; But et al., 2009), *Persicaria ferruginea* (Wedd.) Soják [Polygonaceae] (López et al., 2006), *Persicaria limbata* (Meisn.) H. Hara [Polygonaceae] (Kuete et al., 2014), *Populus canadensis* var. *fremontii* (S. Watson) Kuntze [Salicaceae] and *Populus sargentii* Dode [Salicaceae] (English et al., 1992), *Scutellaria amabilis* H. Hara [Lamiaceae] (Miyaichi et al., 2006), *Scutellaria barbata* D. Don [Lamiaceae]- *Oldenlandia diffusa* (Willd.) Roxb. [Rubiaceae] herb pair (Yang et al., 2018), *Scutellaria indica* L. [Lamiaceae] (Miyaichi et al., 1987), and *Vitex tripinnata* (Lour.) Merr. [Lamiaceae] (Pan et al., 2014). The natural sources of alpinetin were listed in Table 1. The accurate methods for the separation and determination of alpinetin were developed by high-performance liquid chromatography (HPLC), combination of flow injection (FI)-micellar electrokinetic chromatography (MEKC), reverse micelle electrokinetic capillary chromatography (RMEKC), sensitive resonance Rayleigh light scattering (RLS) assay, and liquid chromatography-tandem mass spectrometry (LC-MS/MS) (Liu et al., 2007; Wang et al., 2007; Singh et al., 2015; Bi et al., 2018; Lorençoni et al., 2020).

**TABLE 1:** Natural sources of alpinetin.

Botanical families	Botanical names	Analyzed plant parts	Tested extracts	Quantitative content (mg/g, or %)	References
Asteraceae	*Mikania micrantha* Kunth	Aerial parts	Methanolic extract	0.048 mg/g	Jiang et al. (2001); But et al. (2009)
Combretaceae	*Combretum albopunctatum* Suess	Fruits, leaves	Extracted with CH_2_Cl_2_	1.190 mg/g	Katerere et al. (2004)
Fabaceae	*Dalbergia odorifera* T.C.Chen	Heartwood samples	Methanolic extract	0.011–0.087 mg/g	Zhao et al. (2020)
Fabaceae	*Dalbergia parviflora* Roxb	Dried heartwood	Methanolic extract	0.667 mg/g	Umehara et al. (2008)
Juglandaceae	*Carya cathayensis* Sarg	Leaves	Ethanol extract	-	Cao et al. (2012)
Lamiaceae	*Scutellaria indica* L	Rhizomes	Ethanol extract	1.639 mg/g	Miyaichi et al. (1987)
Lamiaceae	*Scutellaria amabilis* H.Hara	Rhizomes	Methanolic extract	0.667 mg/g	Miyaichi et al. (2006)
Lamiaceae	*Vitex tripinnata* (Lour.) Merr	Leaves, twigs	Extracted with CH_2_Cl_2_	0.087 mg/g	Pan et al. (2014)
Lamiaceae and Rubiaceae	*Scutellaria barbata* D.Don-*Oldenland-ia diffusa* (Willd.) Roxb. herb pair	Dry grass	Deionized water extract	(0.036 ± 0.003) ×10^−3^ mg/g	Yang et al. (2018)
Myrtaceae	*Campomanesia phaea* (O.Berg) Landrum	Leaves	Ethanol extract	-	Lorençoni et al. (2020)
Polygonaceae	*Persicaria ferruginea (Wedd.)* Soják	Leaves	Methanolic extract	0.296 mg/g	López et al. (2006)
Polygonaceae	*Persicaria limbata (Meisn.)* H.Hara	Leaves	-	3.846%	Kuete et al. (2014)
Salicaceae	*Populus canadensis* var. *fremontii (S. Watson)* Kuntze, *Populus sargentii* Dode	Bud exudates	-	Percent of total ion current (0.3)	English et al. (1992)
				Percent of total ion current (<0.1)	
Zingiberaceae	*Alpinia hainanensis* K.Schum	Seeds	Ethanol extract	0.500–1.450%	Rao and Lin (1998)
			Ethanol extract	5.800 ± 0.200 mg/g	Liu et al. (2007)
			Ethanol extract	0.970 mg/g	Wang et al. (2007)
			Ethanol extract	4.843–5.156 mg/g	Zou and Lu. (2011)
			Ethanol extract	4.380–6.710 mg/g	Zhao et al. (2013)
			Methanol extract	1.073–1.463%	Liang et al. (2007)
			Methanol extract	0.140–6.460 mg/g	Liu and Wu. (2009)
			Methanol extract	0.328–0.891%	Li et al. (2010)
			Methanol extract	6.380 mg/g	Bi et al. (2018)
Zingiberaceae	*Alpinia intermedia* Gagnep	Seeds	-	-	Qiao et al. (2001)
Zingiberaceae	*Alpinia mutica* Roxb	Rhizomes	Crude chloroform extract	1.327 mg/g	Malami et al. (2016); Malami et al. (2017a)
Zingiberaceae	*Alpinia pinnanensis* T.L.Wu & S.J.Chen	Rhizomes	Crude methanolic extract	6.308 mg/g	Giang et al. (2005)
Zingiberaceae	*Amomum subulatum* Roxb	Seeds	Extracted with Et_2_O	−0.011–0.016%	Rao et al. (1976)
			Methanolic extract		Singh et al. (2015)
Zingiberaceae	*Boesenbergia rotunda* (L.) Mansf	Rhizomes, leaves, ste-ms	Methanolic extract	0.081–3.738 mg/g	Tan et al. (2015); Morikawa et al. (2008); Nguyen et al. (2020)
		Rhizomes	Crude ethanolic extract	1.730%	Wangkangwan et al. (2009)
		Rhizomes	Crude ethanolic extract	6.522 mg/g	Tewtrakul et al. (2003)
		Transgenic B. rotunda cells	Methanolic extract	0.927 mg/g	Liew et al. (2020)

**FIGURE 2:**
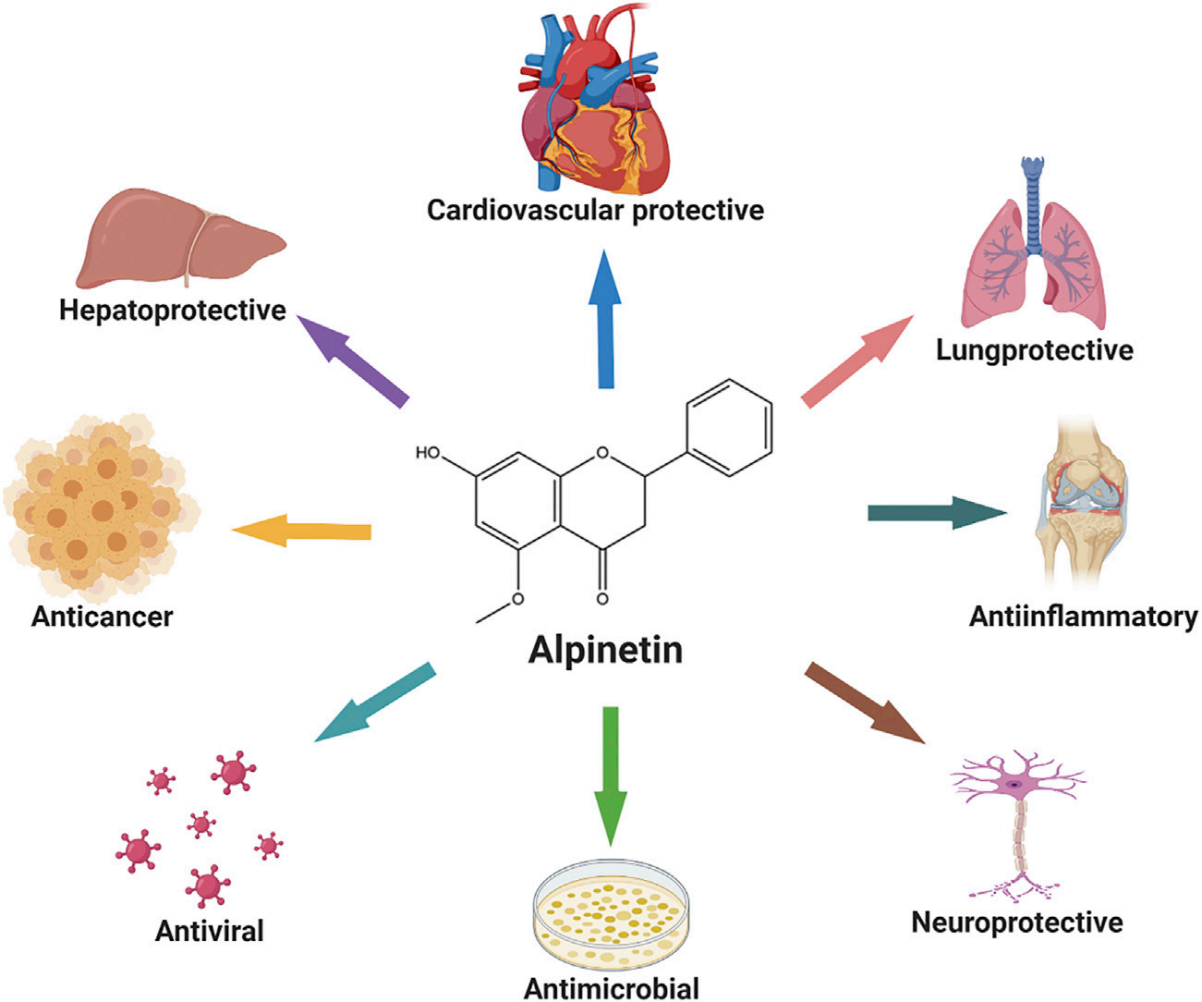
The main pharmacological activities of alpinetin.

Alpinetin is the major component of Chinese patent drugs such as Jianweizhitong tablet, Fufangcaodoukou tincture, Baikoutiaozhong pill, and Xingqiwenzhong granule, which have been used clinically in the treatment of digestive disorders, including epigastric pain, belching, nausea, vomiting, and anorexia (Gan, 2005; Huang, 2016; Chen et al., 2018; Xiang et al., 2019). Abundant researches have been performed and centralized on the pharmacological activities of alpinetin, and elucidated its prospective potential for carcinoma (Wang P et al., 2017; Zhang et al., 2020), inflammatory diseases, (Huo et al., 2012), bacterial infection (Huang et al., 2006), virus infection (But et al., 2009), liver injury (Liu T. G et al., 2019), cardiovascular diseases (Jantan et al., 2004), and neuro disorders (Liu E. Y. L et al., 2019) associated with modulating multiple signaling pathways (Figure 2). Additionally, pharmacokinetic assessment has become vitally important for estimating and optimizing clinical efficacy of drugs (Walker, 2004). The pharmacokinetic profiles of this compound have been investigated to explore its biological feature in the body. For example, alpinetin was subjected to profound first-pass glucuronidation as a flavonoid with one hydroxyl group, and exhibited poor oral bioavailability (Qiu et al., 2019). Besides, alpinetin was previously reported to include low systemic toxicity properties, which may be determined by its metabolism (He et al., 2006; Costa et al., 2014).

At present, the systemic overview on the pharmacological activities and pharmacokinetics features of alpinetin is absent. Thereby, this article comprehensively summarized these properties that have been reported to date, and devoted to provide scientific basis of alpinetin for the development of new drugs and assess its future research opportunities. The literature searches (1976–2021) were conducted in electronic databases such as PubMed, Web of Science, Elsevier, Springer, Wiley, Science Direct, ACS Publications, China National Knowledge Infrastructure, Google Scholar, Scopus, The Plant List Database and related scientific journals. Key words utilized for the systematic searches were: “Alpinetin”, “Flavonoids”, “Mechanisms of action”, “Pharmacokinetics” and “Pharmacology”. The publications and further relevant papers featured in this review were screened and optimized based on the themes as follows: natural sources, pharmacological activities, and pharmacokinetics profiles along with concerned improvement strategies. The ultimately yielded studies regarding alpinetin in different languages were carefully checked and completely cited.

## Pharmacological Activities

### Anticancer Activity

**FIGURE 3:**
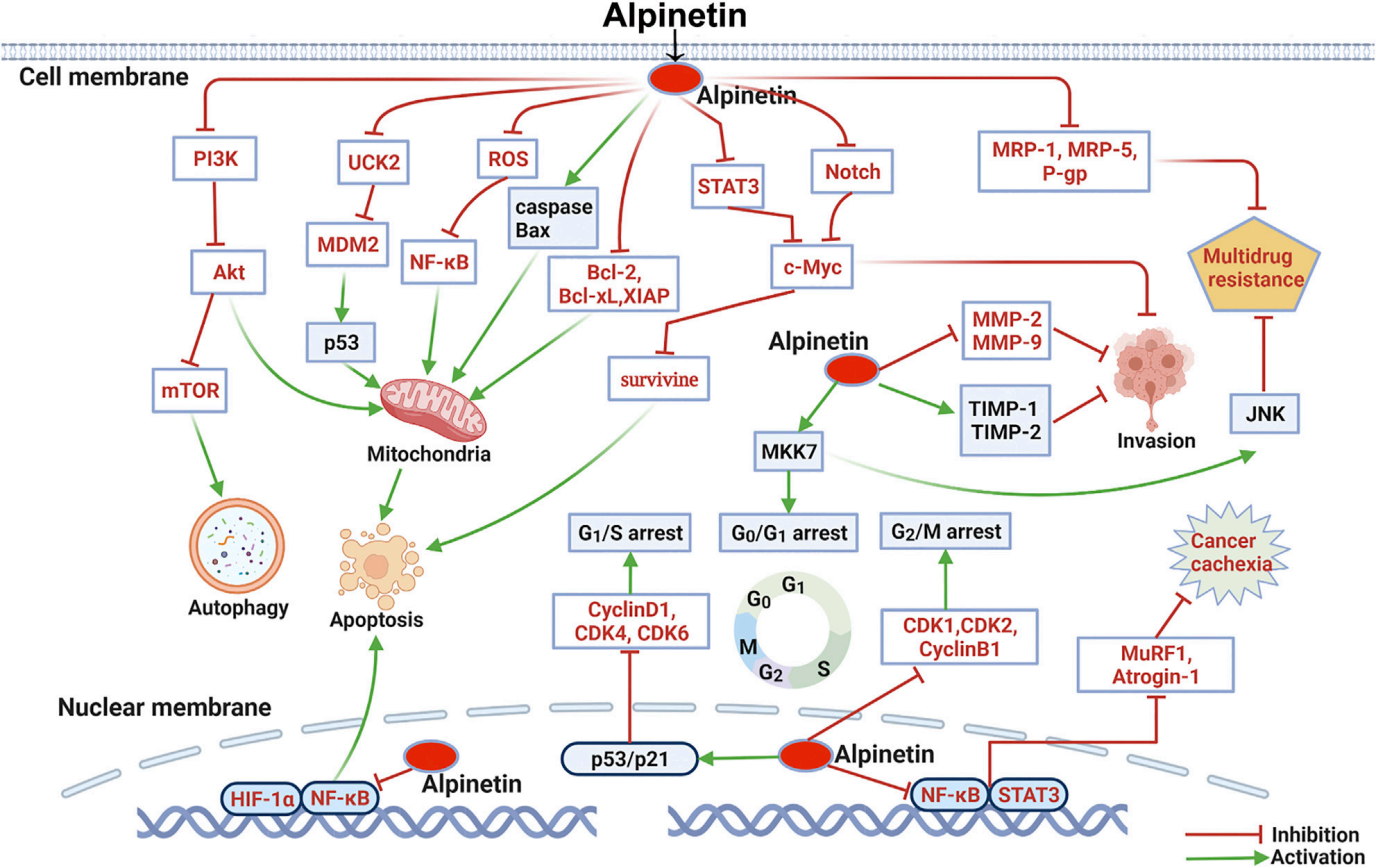
Molecular pathways involved in the anti-cancer activities of alpinetin.

Cancer is one of the most critical and prevalent health problems in late years, characterized by hyperproliferative disorder induced through the dysfunction of numerous important genes, metabolism and signaling (Millimouno et al., 2014). Natural flavonoids are important sources of antitumor drugs. Numerous studies on alpinetin have illustrated its anticancer properties against a wide variety of human carcinoma cell lines including 4T1, MCF-7 and MDA-MB-231 breast tumor cell lines (Zhang et al., 2020), A549, NCI-H460, SK-MES-1, NCI-H292 and A549/cis-diammined dichloridoplatium (CDDP) lung tumor cell lines (Wu et al., 2015; Hou et al., 2021), SKOV3 and OVCAR-8 ovarian cancer cell lines (Zeng et al., 2018; Zhao et al., 2018), TCA-8113 and CAL-27 tongue squamous carcinoma cell lines (Guo et al., 2020), U-87, U-251 and U-373 Glioma cell lines (Wang et al., 2016), HepG2 hepatic tumor cell line (Tang et al., 2012), BxPC-3, PANC-1 and AsPC-1 pancreatic carcinoma cell lines (Du et al., 2012), AGS and N87 gastric cancer cell lines (Wang et al., 2013), HT-29 and HCT116 colon cancer cell lines (Malek et al., 2011; Malami et al., 2017a), Hela cervical carcinoma cell line (Cao et al., 2012), CCRF-CEM and multidrug-resistant P-glycoprotein over-expressing CEM/ADR5000 leukemia cell lines (Kuete et al., 2014), and EC9706 esophageal cancer cell line (Tang et al., 2021). Furthermore, alpinetin could suppress the growth of mouse Lewis lung carcinoma cells (LLC) (Hou et al., 2021) and N1-S1 hepatic cancer cells (Tang et al., 2012). The therapeutic effects of alpinetin on various carcinoma cells through multiple molecular mechanisms were summarized as follows (Table 2 and Figure 3).

**TABLE 2:** Pharmacological effects of alpinetin.

Pharmacological effects	Cell lines/Model	Application	Doses/Duration	Effects/Molecular mechanisms	References
Anticancer activity	4T1, MCF-7	*In vitro*	25–100 μM for 24 or 48 h	caspase-9↑, caspase-3↑, PARP↑, Bax↑, Bcl-2↓	Zhang et al. (2020)
				cytochrome-c↑, p-p65↓, p-IκBα↓, HIF-1α↓	
	MDA-MB-231	*In vivo*	100 mg/kg, i.p., tiw, for 28 days	caspase-3↑, PARP↑, Bax↑, Bcl-2↓, p-p65↓	Zhang et al. (2020)
				p-IκBα↓	
	LLC	*In vivo*	5 and 10 mg/kg, i.p., daily, for 3 weeks; 25 and 50 mg/kg, i.g., daily, for 2 weeks	MuRF1↓, Atrogin-1↓, CDK6↓, CyclinD1↓	Hou et al. (2021)
				FASN↓, SCD↓, CD11b↓, F4/80↓, CD163↓	Zhang et al. (2021)
				IL-10↓, CCL-2↓, CCL-7↓	
	NCI-H460	*In vitro*	30 μM for 24 h	CDK6↓, CyclinD1↓, FASN↓, SCD↓, CD11b↓	Hou et al. (2021)
				F4/80↓, CD163↓, IL-10↓, CCL-2↓, CCL-7↓	
	A549, SK-MES-1	*In vitro*	50–400 μM for 24, 48 or 72 h	caspase-9↑, caspase-3↑, caspase-8↑, cytochrome-c↑, Bax↑, Bcl-2↓, XIAP↓, Akt↓, PI3K↑	Wu et al. (2015)
	NCI-H292				
	A549/(CDDP)	*In vivo*	50 mg/kg, i.p., weekly for 4 weeks	MRP-1↓, MRP-5↓, P-gp↓	Wu et al. (2015)
	OVCAR-8	*In vitro*	25–400 μM for 24, 48, or 72 h	p-STAT3↓, c-Myc↓, survivin↓, caspase-3↑, caspase-9↑, Bax↑, Bcl-2↓	Zeng et al. (2018)
	Hela	*In vitro*		IC_50_ (>100 μM)	Cao et al. (2012)
	SKOV3	*In vitro*	50–400 μM for 48 h	caspase-3↑, Bax↑, Bcl-2↓, PARP↑, CyclinD1↓, CDK4↓, CDK6↓, TIMP-1↑, TIMP-2↑, MMP-2↓, MMP-9↓, p-STAT3↓, c-Myc↓, survivin↓	Zhao et al. (2018)
	TCA-8113	*In vivo*	50 mg/kg, i.p., once every 2 days, for 12 days	miR-211-5p↑	Guo et al. (2020)
	CAL-27	*In vitro*	100–500 μM for 24 h	p-p53↑, p21↑, PARP↑, CyclinD1↓, miR-211-5p↑, NICD↓, HES1↓	Guo et al. (2020)
	U-87	*In vivo*	25, 75 mg/kg, i.v., once every 2 days, for 2 weeks	Cleaved Notch1↓	Wang et al. (2016)
	U-251, U-373	*In vitro*	20–80 μM for 48 h	HES↓, c-Myc↓, Cleaved Notch1↓	Wang et al. (2016)
	HepG2	*In vitro*	20–80 μg/ml for 24, 36 or 48 h	p-MKK7↑	Tang et al. (2012)
	HCT116	*In vitro*		IC_50_ (39.6 μg/ml)	Malek et al. (2011)
	BxPC-3, PANC-1, AsPC-1	*In vitro*	20–80 μg/ml for 24, 48, or 72 h	caspase-9↑, caspase-3↑, caspase-8↑, Bax↑, Bcl-2↓, Bcl-xL↓, XIAP↓, cytochrome-c↑	Du et al. (2012)
	AGS, N87	*In vitro*	40–160 μM for 24, 48, or 72 h	Bax↑, Bcl-2↓, cyto-c↑, caspase-9↑, caspase-3↑, CDK1↓, CDK2↓, CyclinB1↓	Wang et al. (2013)
	CEM; CEM/ADR5000	*In vitro*		IC_50_ (88.22 ± 8.78 μM); IC_50_ (116.07 ± 7.93 μM)	Kuete et al. (2014)
	HT-29	*In vitro*	6.25–400 μM for 48 or 72 h	UCK2↓, MDM2↓, p53↑, caspase-3↑, Bax↑, Bcl-2↓, cytochrome-c↑	Malami et al. (2017a)
	EC9706	*In vitro*	25–100 μM for 48 h	caspase-9↑, caspase-3↑, Ki67↓, PCNA↓, Beclin1↑, ATG8↑, LC3II↑, p-PI3K↓, p-Akt↓, p-mTOR↓	Tang et al. (2021)
Anti-inflammatory activity	LPS-induced mouse acute lung injury	*In vivo*	50 mg/kg, i.p., 1 h prior to administration of LPS	TNF-α↓, IL-6↓, IL-1β↓	Huo et al. (2012)
	RAW 264.7	*In vitro*	25 μM for 24 h	TNF-α↓, IL-6↓, IL-1β↓, p-IκB↓, p-p65↓, p-ERK↓, p-p38↓, PPAR↑, DNMT3A↑	Yu et al. (2020)
			80–240 μg/ml for 24 h		Huo et al. (2012)
			50–1,000 μg/ml for 24 h		Hu et al. (2020)
	LPS-induced mouse mastitis	*In vivo*	10–50 mg/kg, i.p	MPO↓, TNF-α↓, IL-6↓, IL-1β↓, TLR4↓, p-IκB↓, p-p65↓	Chen et al. (2013)
	DSS-induced mice colitis	*In vivo*	25–100 mg/kg, i.g., daily, for 7 days	TNF-α↓, IL-6↓, IL-1β↓, TLR4↓, p-IκB↓, p-p65↓, MPO↓	He et al. (2016)
	THP-1	*In vitro*	50–200 μg/ml	NLRP3↓, ASC↓, caspase-1↓, TNF-α↓, IL-6↓, IL-1β↓, TLR4↓, p-IκB↓, p-p65↓, p-JNK↓, p-ERK↓, p-p38↓, PPAR-γ↑	He et al. (2016)
					Hu et al. (2013)
	DMM-induced mice osteoarthritis	*In vivo*	1 mM, intra-articular knee injection daily for 4 days	COL2A1↑, ADAMTS-5↓, MMP-13↓, BCL-2↑, CDK1↑, p-IκBα↓, p-ERK↑	Gao et al. (2020)
	HPMECs	*In vitro*	40–320 μg/ml for 48 h	ICAM-1↓, TNF-α↓, AQP-1↑	Wang et al. (2017)
	Mouse T lymphocytes	*In vitro*	20–60 mg/ml for 48 h	IL-2↓, IFN-γ↓, IL-4↓, IL-6↓, p-IκB↓, p65↓	Guan et al. (2014)
	LPS-induced mouse chondrocytes damage	*In vitro*	0.3125–50 mg/ml for 24 h	COL2A1↑, IL-1β↓, IL-6↓, iNOS↓, TNF-α↓, MMP-13↓	Dai et al. (2020)
	LPS-induced mouse endometritis	*In vivo*	10–40 mg/kg, i.p., 1 h before LPS treatment	MPO↓, TNF-α↓, IL-6↓, IL-1β↓, TLR4↓, p-IκB↓, p-p65↓, PPAR-γ↑	Liang et al. (2018)
	Carrageenan induced acute inflammation	*In vivo*	10–40 mg/kg, i.g., daily, for 7 days	MPO↓, TNF-α↓, IL-1β↓, PPAR-γ↑, p-p65↓	Cui et al. (2019)
	Severe acute pancreatitis caused acute lung injury	*In vivo*	40–320 μg/ml, i.g., for 6, 12, 24 h	AQP-1↑, TNF-α↓	Liang et al. (2016)
	Cecal ligation and puncture induced sepsis rats	*In vivo*	40–160 mg/kg, i.p., for 24 h	MIP-2↓, TNF-α↓, IL-18↓, IL-10↑, MPO↓, SOD↑, MDA↓, GSH↑, p-AKT↑, Nrf2↑, HO-1↑	Ren et al. (2021)
	Ovalbumin-induced allergic asthma mice	*In vivo*	25–100 mg/kg, i.p., daily on days 21–23	IgE↓, IL-4↓, IL-5↓, IL-13↓, p-p65↓, p-IκB↓, p-Akt↓, p-PI3K↓, HO-1↓	Wu et al. (2020)
	COPD in rat model	*In vivo*	20 mg/kg, i.g., daily, for four consecutive weeks	TNF-α↓, IL-6↓, IL-10↑, caspase-9↓, caspase-3↓, TGF-β1↓, α-SMA↓	Su et al. (2020)
	DSS-induced mouse colitis	*In vivo*	7.5–30 mg/kg, i.g., daily, for 10 days	MPO↓, TNF-α↓, IL-1β↓, IL-17↓, IL-10↑, RORγt↓, Foxp3↑, CYP1A1↑, AhR (cytosol)↓, AhR (nuclear) ↑, ARNT↑, miR-302↑, DNMT-1↓	Lv et al. (2018)
			25–100 mg/kg, i.p., daily, for 7days	CREB↑, Occludin↑, ZO-1↑, Claudin-2↓, MDA↓, SOD↑, MPO↓, Nrf2↑, HO-1↑	Tan and Zheng. (2018)
	Caco-2, NCM460	*In vitro*	3–30 μM for 24 h	Claudin-7↑, Occluding↑, E-cadherin↑, caspase-3↓, LC3B↑, Beclin-1↑, Atg5↑, Atg7↑, p-RPS6↓, p-p70S6K↓, AhR↑, suv39h1↑, TSC2↑, PTEN↓, p-ERK↓, p-AMPKα↓, p62↓	Miao et al. (2019)
	CLP-induced PICS	*In vivo*	50 mg/kg, intravenously infu-sed, daily, for 8 days	TNF-α↓, IL-6↓, CD4^+^ T↑, CD8^+^ T↑, MPO↓, ROS↓, SOD↑	Liu et al. (2021)
	DSS-induced colitis in mice	*In vivo*	50 mg/kg, i.g., daily, for 9 days	MPO↓, TNF-α↓, IL-6↓, iNOS↓, ICAM-1↓, MCP-1↓, COX-2↓, IFNγ↓, IL-1β↓, IL-1α↓, Cyp3a11↑, Mdr1a↑, PXR↑	Yu et al. (2020)
Hepatoprotective activity	LPS/D-Gal-induced mouse liver injury	*In vivo*	12.5–50 mg/kg, i.p., 1 h before LPS/D-gal treatment	ALT↓, AST↓, MDA↓, MPO↓, TNF-α↓, IL-1β↓, p-IκB↓, p-p65↓, Nrf2↑, HO-1↑	Liu et al. (2019)
	Hepatic ischemia/reperfu-sion injury in mouse	*In vivo*	50 mg/kg, i.p., 1 h before ischemia	ALT↓, AST↓, LDH↓, TNF-α↓, IL-1β↓, IL-8↓, MCP-1↓, BAX↓, BCL2↑, caspase-3↓, p-p65↓, p-IKKβ↓, Iκbα↑, p-p38↓, p-JNK↓	Pan et al. (2021)
	Carbon tetrachloride indu-ced mouse liver fibrosis	*In vivo*	15 and 60 mg/kg, i.p., daily, for last 4 weeks	ALT↓, AST↓, LDH↓, Hydroxyproline↓, α-SMA↓, Fibronectin↓, α1(I) procollagen↓, TNF-α↓, IL-1β↓, IL-6↓, IL-10↑, MDA↓, GSH↑, CAT↑	Zhu et al. (2021)
				GSH-Px↑, SOD↑, VEGF↓, PDGF↓, HIF-1α↓, VEGFR2↓, PDGF-βR↓, NLRP3↓, Caspase-1↓, ASC↓, IL-18↓, GCLC↑, HO-1↑, NQO1↑, GCLM↑, Nrf2↑	
	High fat diet-induced	*In vivo*	12.5–50 mg/kg, i.g., daily, for 8 weeks	ALT↓, AST↓, SOD↑, CAT↑, GPx↑, MDA↓, XO↓, HO-1↑, Nrf2↑, XO↓, TXNIP↓, TNF-α↓, IL-1β↓, IL-4↓, IL-6↓, IL-17↓, TLR4↓, p-IκBα↓, p-NF-κB↓, SCD1↓, FAS↓, PPARα↑, SREBP-1c↓, LXR-α↓, ELOVL-2↓, p-JNK↓, p-IRS1↑	Zhou et al. (2018)
	NAFLD				
Cardiovascular protective activity	Rabbit platelets	*In vitro*	1.8–18.2 μg/ml	IC_50_ (41.6 ± 2.7 μM)	Jantan et al. (2004)
				Platelet-activating factor antagonistic activities	
	Coronary heart disease rat model	*In vivo*	40–160 mg/kg, i.g., daily, for 4 weeks	LVEDV↓, SV↑, TG↓, TC↓, HDL-C↑, LDL-C↓, NO↑, ET-1↓, PGI2↑, TNF-α↓, MCP-1↓, ICAM-1↓, p-ERK↓, p-MEK↓	Dai et al. (2021)
	THP-1, HMDMs	*In vitro*	50–150 μg/ml for 24 h	PPAR-γ↑, LXR-α↑, ABCA1↑, ABCG1↑	Jiang et al. (2015)
	Rat myocardial cells	*In vitro*	40–120 mg/ml for 48 h	Activates δ receptor, PKC↑, ERK↑, Bcl-2↑, Bax↓, caspase-9↓, caspase-3↓	Suo et al. (2014)
	Mouse VSMC	*In vitro*	10^−7^–10^–9^ mol/L for 12 h	NO↓, LDH↓	Li and Du. (2004)
	Mouse mesenteric artery	*In vitro*	10–100 μM	Inhibition of Ca2^+^ influx, NO↑, PKC↓	Wang et al. (2001)
Antimicrobial activity	*Helicobacter pylori*	*In vitro*	for 3 days	MIC (1.25 μg/ml)	Huang et al. (2006)
	*Escherichia coli*	*In vitro*	for 16–18 h	MIC (>3.85 mg/ml)	Huang et al. (2006)
	*Salmonella typhi*	*In vitro*	for 16–18 h	MIC (3.85 mg/ml)	Huang et al. (2006)
	*Klebsiella* penumoniae	*In vitro*	for 16–18 h	MIC (3.85 mg/ml)	Huang et al. (2006)
	*Pseudomonas* pyocyanea	*In vitro*	for 16–18 h	MIC (3.85 mg/ml)	Huang et al. (2006)
	*Enterobacter* aerogenes	*In vitro*	for 16–18 h	MIC (3.85 mg/ml)	Huang et al. (2006)
	*Pseudomonas maltophilia*	*In vitro*	for 16–18 h	MIC (3.85 mg/ml)	Huang et al. (2006)
	*Citrobacter* diversus	*In vitro*	for 16–18 h	MIC (3.85 mg/ml)	Huang et al. (2006)
	*Pseudomonas cepacia*	*In vitro*	for 16–18 h	MIC (1.925 mg/ml)	Huang et al. (2006)
	Drug-resistant *Aeromonas* hydrophila	*In vitro*	for 24 h	MIC (128–256 μg/ml)MBC (512–1,024 μg/ml)	Chen et al. (2021)

Antiviral activity	Respiratory syncytial virus	*In vitro*		IC_50_ (77.0 μM), TI (6.0)	But et al. (2009)
	Parainfluenza type 3	*In vitro*		IC_50_ (154.4 μM), TI (3.0)	But et al. (2009)
	influenza type A	*In vitro*		IC_50_ (308.5 μM), TI (1.5)	But et al. (2009)
	HIV-1 protease	*In vitro*		IC_50_ (>100 μg/ml)	Tewtrakul et al. (2003)
	HIV-1 infectived HOG·R5	*In vitro*		IC_50_ (130 μM)	Pan et al. (2014)
	SARS-CoV-2	*In vitro*		Docked into the active site pocket of SARS-CoV-2 M^pro^, binding energy (-7.51 kcal/mol), inhibition constant (3.12 μM)	Gurung et al. (2021)
Neuroprotective activity	PC12 cells	*In vitro*	1–10 μM for 48 h	AChE↑, NF68↑, NF200↑	Liu et al. (2019)
Antioxidant activity	UV radiation	*In vitro*		Displayed an extensive absorption in the extent of harmful UV radiation (270–390 nm)	Shireen et al. (2017)

↑, Up-regulation or activation; ↓, Down-regulation or inhibition.

#### Induction of Cancer Cell Apoptosis

Apoptosis, an extremely controlled and regulated process, has important functions in the development and treatment of tumors (Ameisen, 2002; Gerl and Vaux, 2005; Elmore, 2007). Apoptosis primarily processes via the mitochondrial (intrinsic) and the death receptor-mediated (extrinsic) molecular transduction pathways. Interior and exterior apoptotic pathways motivate caspases-9/8, which subsequently activate caspases-3, PARP, and finally lead to the evolution of cell apoptosis body (Putcha et al., 2002; Martinvalet et al., 2005).

Previous investigations showed that alpinetin (40–160 μM) was certified to transform the membrane potential of mitochondrial, which resulted in cytochrome c delivering and caspase inciting, and causing apoptosis in human gastric carcinoma AGS and N87 cells (Wang et al., 2013). Likewise, alpinetin concentration-dependently restrained cell proliferation and provoked apoptosis in BxPC-3 and A549 cells (Du et al., 2012; Wu et al., 2015). The mechanisms involved in intrinsic and extrinsic apoptotic pathways through elevating protein production of Bax, caspases-3, caspases-8 and caspases-9, inhibiting the expression levels of Bcl-2, Bcl-xL and XIAP, and promoting the release of cytochrome c to the cytoplasm. Afterwards, Zhao et al. assessed the apoptosis of SKOV3 cells activated by alpinetin. The results described that administrated with alpinetin (50–400 μM) noticeably enhanced the expression of Bax, caspase-3 and PARP, and reduced Bcl-2 secretion by inhibiting the STAT3 signaling pathway in a dose and time dependent manner (Zhao et al., 2018). In addition, p53 signaling pathway is a crucial regulator of cell cycle arrest and apoptosis (Zhang and Lu, 2009). Studies have suggested that alpinetin (6.25–400 μM) specifically targeted to the uridine-cytidine kinase 2 (UCK2) enzyme involved in gene synthesis, and induced p53 dependent mitochondrial apoptosis in HT-29 cells (Malami et al., 2016; Malami et al., 2017a). Recently, Zhang et al. demonstrated that alpinetin (at dose of 25–100 μM) induced mitochondria associated apoptosis in 4T1 and MDA-MB-231 cells through excitation of caspase-3, caspase-9 and PARP, remarkably boosting the ratio of Bax to Bcl-2, and stimulating cytochrome c release from mitochondria to the cytoplasm (Zhang et al., 2020). Furthermore, Tang et al. unveiled that alpinetin significantly induced apoptosis and autophagy in EC9706 cells. The administration of alpinetin (25–100 μM) boosted caspase-3, caspase-9, Beclin1 and ATG8 expressions, elevated the ratio of LC3II/LC3I, and reduced Ki67, PCNA, and P62 generation by suppressing PI3K/Akt/mTOR signaling pathway (Tang et al., 2021).

Therefore, alpinetin may be available in inducing cytotoxicity against diverse types of carcinoma cells through the enablement of apoptosis. Definitely, we acknowledge that pyroptosis and ferroptosis are the other major cell death patterns (Chen X et al., 2021). Further experiments are required to monitor molecular mechanisms of alpinetin regulating these cell death patterns in tumor cells, and to detect potential therapeutic markers in the prevention and treatment of cancers.

#### Inhibition of Invasion and Metastasis of Cancer Cells

Tumor invasion and metastasis, the main characteristics of tumor biology, are the critical hurdles in the prolific treatment of cancers (Kang et al., 2015). Matrix metalloproteinase (MMPs) and tissue inhibitor of metalloproteinase (TIMPs) are the crucial factors for tumor invasion (Sun et al., 2016; Jia et al., 2017). MMPs stimulate tumor invasion, whereas TIMPs behave as inhibitors of MMPs. *Alpinia hainanensis* K. Schum. [Zingiberaceae] was previously tested to possess anti-migratory and anti-invasion properties handling with HT-1080 cells (Park et al., 2013). As a natural flavonoid amply existed in the seeds of *Alpinia hainanensis* K. Schum. [Zingiberaceae], alpinetin (20–80 μM) could also evidently attenuated the invasion of U-87, U-251, and U-373 cells by suppressing Notch pathway (Wang et al., 2016). Afterwards, Zhao et al. confirmed that alpinetin obviously suppressed the migration of SKOV3 ovarian tumor cells via decreasing MMP-2 and MMP-9 expression levels and improving TIMP-1 and TIMP-2 secretion (Zhao et al., 2018). Similarly, after treated with 100 μM alpinetin for 24 h, the migration capacity of OVCAR-8 cells was repressed related to the inhibition of STAT3/c-Myc axis (Zeng et al., 2018). In a recent study, the wound-scraping assay of 4T1 and MDA-MB-231 cells also illustrated that alpinetin markedly inhibited the migration of human breast tumor cells (Zhang et al., 2020). Furthermore, Aminopeptidase N (APN) activation is of great importance to tumor metastasis. Morikawa et al. isolated and evaluated alpinetin for the inhibitory effect on APN activity. The data revealed that alpinetin (30 μM) showed a potent restriction effect measured to 36.4%, and could be applied as an APN inhibitor to abrogate tumor metastasis (Morikawa et al., 2008).

These outcomes indicate that alpinetin seemes to be effective in regressing the metastasis of tumor cells. Nevertheless, the premise of assessing anti-metastasis activities of alpinetin in added neoplasm models is to conduct more studies and probe auxiliary mechanisms.

#### Induction of Cancer Cell Cycle Arrest

Cell cycle arrest, regulated by a class of enzymes (cyclin-dependent kinases, CDKs), is considered as one of the major causes for carcinoma cell death (Asghar et al., 2015; Benada and Macurek, 2015). Tang et al. reported for the first time that alpinetin (60 μg/ml) caused human hepatic tumor cells arrested in the G_0_/G_1_ phase. The results elucidated that alpinetin hampered HepG2 cells cycle progression through activation of p-MKK7, whereas the arrested effect was reversed by siRNA targeted on MKK7 (Tang et al., 2012). Analogically, flow cytometry explored that alpinetin treatment (40–60 μg/ml) dramatically stimulated cell cycle arrest in the G_0_/G_1_ phase in BxPC-3, PANC-1 and AsPC-1 cells (Du et al., 2012). Whereafter, Wang et al. evaluated that alpinetin (40–160 μM) evidently lowered CDK1, CDK2 and cyclin B1 expressions, and provoked the cell cycle of AGS and N87 cells arrested in the G_2_/M phase (Wang et al., 2013). Zhao et al. also illustrated that alpinetin remarkably decreased CyclinD1, CDK4 and CDK6 production, and arrested SKOV3 cells in the G_1_ phase (Zhao et al., 2018). Furthermore, Guo et al. suggested that alpinetin (100–500 μM) inhibited OSCC cells proliferation and arrested cells in the G_1_ phase via upregulating miR-211-5p level and disturbing the Notch pathway (Guo et al., 2020). The latest research executed by Hou et al. likewise reported that alpinetin administration (30 μM) notably decreased the transcription of CDK6 and CyclinD1, and hindered cell cycle progression in A549 and NCI-H460 cells (Hou et al., 2021).

According to the aforesaid reports, alpinetin is found to induce the G_0_/G_1_ and G_2_/M phases of the cell cycle arrested through modulating CDKs, thus generate antiproliferative effects in various tumor cells. Alpinetin may act as a cell cycle nonspecific agent for future tumor treatment.

#### Reversion of Multidrug Resistance

Multidrug resistance-associated protein (MRP) and P-glycoprotein (P-gp) play important roles in drug resistance, which has become an inevitable issue for successful chemotherapy (Wesolowska, 2011; Ma et al., 2014). As reported, after administrated with alpinetin at 60 µg/ml and CDDP at 20 µg/ml for 24 h, alpinetin strongly boosted the chemosensitivity of HepG2 hepatoma cells to CDDP. The results found that co-administered with alpinetin and CDDP exerted a synergy effect for suppressing HepG2 cell proliferation and growth. The efficacy was identified with MKK7/JNK transduction pathway enabling (Tang et al., 2012). Afterwards, Kuete et al. appraised the antiproliferative activity of alpinetin toward CCRF-CEM and multidrug-resistant P-glycoprotein over-expressing CEM/ADR5000 leukemia cells. The recorded IC_50_ values being 88.22 ± 8.78 μM and 116.07 ± 7.93 μM, respectively. The degree of resistance was 1.32-fold, suggesting that alpinetin could be available to turn over multidrug-resistant in leukemia cells (Kuete et al., 2014). Furthermore, therapeutic doses of alpinetin (50 mg/kg) overturned the drug fastness to CDDP (2 mg/kg) in A549/CDDP cells by restraining MRP-1, MRP-5 and P-gp generation, and restoring the sensitivity of cells to CDDP (Wu et al., 2015). Interesting, the tumor volume was also apparently smaller in alpinetin combination with CDDP treated group than in the group administrated with CDDP alone from 14 days after vaccination. Therefore, alpinetin is worthy of further exploration for the therapeutic regimen of malignant tumors, either as a promising chemosensitizer or adjuvant. Combined alpinetin with CDDP or other chemotherapeutic drugs may bring about more beneficial opportunities to treat malignant tumors.

#### Anti-Cancer Cachexic

Cancer cachexia, featured with a polyfactorial musculi skeleti loss syndrome, has been illustrated to bring serious untoward effects while antitumor agents are adhibited (Fearon et al., 2013). Administrated with 25 and 50 mg/kg alpinetin conspicuously mitigated LLC-acquired medium-activated C2C12 depauperating myotube, decreased Atrogin-1 and MuRF1 levels in cachectic muscle, reduced muscular dystrophy in LLC tumor-bearing mice, and inhibited cancer cachexia in a dose-dependent manner. Meanwhile, the underlying molecular mechanisms have been evaluated and revealed that alpinetin obviously promoted PPARγ expression, thereby restraining NF-κB and STAT3 activation both *in vitro* and *in vivo* (Zhang et al., 2021). Importantly, the researchers were taking the lead, and initially afforded new perceptions of alpinetin against cancer cachexia ascribed to PPARγ activation. The portion of the alpinetin effect on cancer cachexia related with other molecule mechanisms needs to be further quested.

Overall, through disparate signaling mechanisms, alpinetin is effective in modulating genes and proteins correlated to the control of cancer cell apoptosis, autophagy, invasion, metastasis, cell cycle arrest, multidrug resistance, and cancer cachexic, suggesting that it may be a promising agent for cancer treatment. Since tumor immunotherapy has cumulatively become successful curative strategies for the treatment of cancers in preclinical patterns and clinic trials (Baxevanis et al., 2009), the competence of alpinetin to initiate an alternative and vigorous host immune reaction against cancer cells is a crucial new opportunity in the field of oncology.

#### Anti-Inflammatory Activity

**FIGURE 4:**
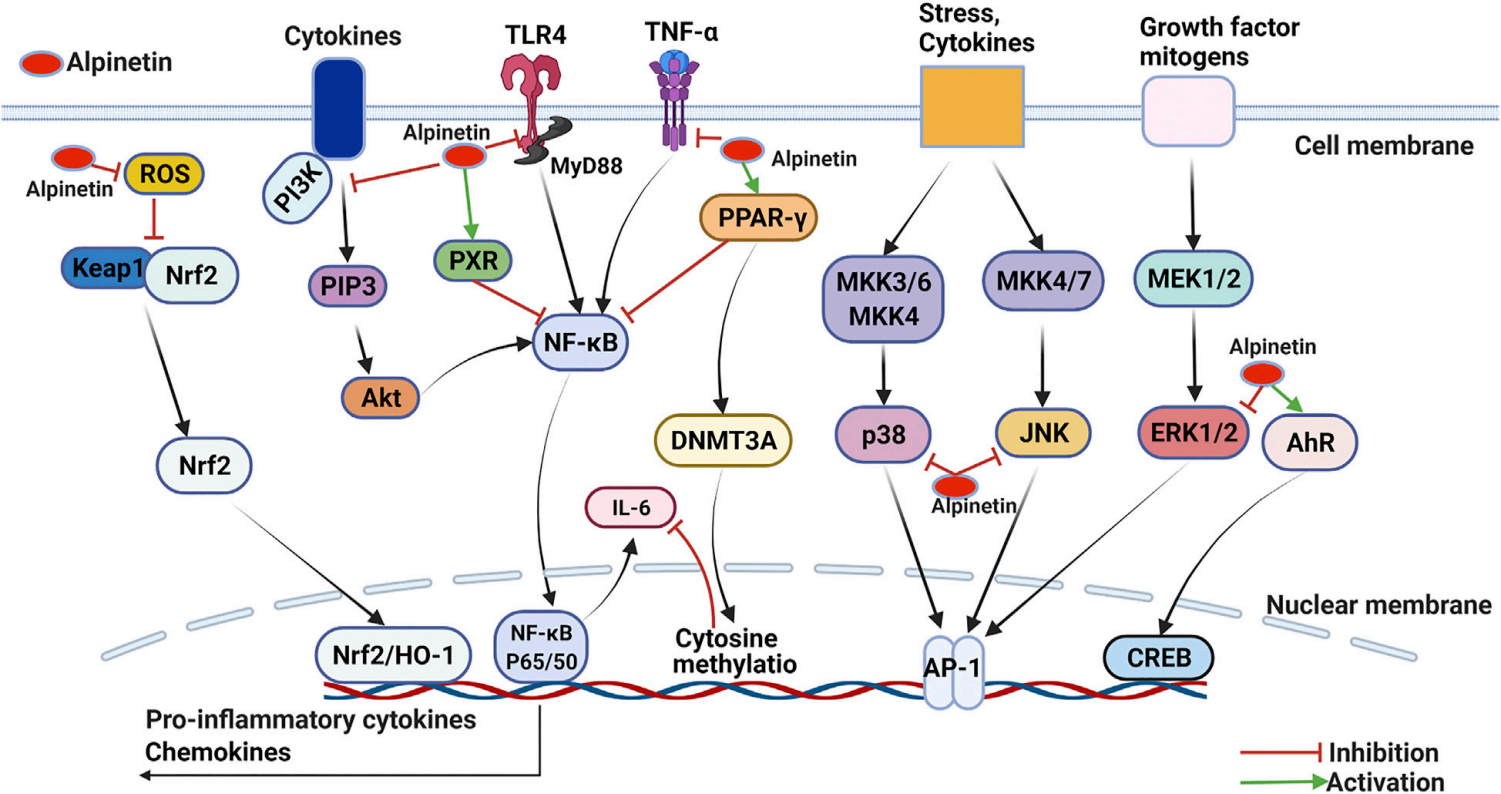
Molecular pathways involved in the anti-inflammation activities of alpinetin.

Inflammation is an accommodative response encompassing vital phylactic function in the organic immunity system against harmful states comprising physiological damage and infection. Chronic or immoderate inflammation are responsible for many disorders of the host, such as pneumonia, cancer, dementia, auto-immune ailments, multiple sclerosis, etc. (Xu and Larbi, 2018; Pu et al., 2020). Immune cells are firstly activated by injurious stimuli in the pathological processes of inflammation. Subsequently, various inflammatory mediators are secreted with multiple signal transduction pathways enabled, which ultimately contribute to partial or general inflammatory injuries (Varela et al., 2018). Alpinetin is capable of inhibiting various inflammatory disorders and exhibiting potential therapeutic effects through numerous signaling mechanisms (Table 2 and Figure 4).

#### Inhibition of NF-κB

Nuclear factor-κB (NF-κB) is a family of transcription factors, and has long been considered the central mediator of the process in inflammation and immunity (Kim et al., 2017; Zhang et al., 2017). TLR4 is one of the most critical upstream signaling axis molecules for NF-κB pathway activation, which in turn manage the secretion of varieties of proinflammatory cytokines, chemokines, and adhesion molecules (Yoshimura, 2006; Gong et al., 2021).

Huo et al. originally appraised that alpinetin markedly downregulated tumor necrosis factor-α (TNF-α), interleukin-1β (IL-1β), and interleukin-6 (IL-6) expression levels in lipopolysaccharide (LPS)-irritated RAW 264.7 macrophages *in vitro* and LPS-provoked acute lung injury mice model *in vivo*. Alpinetin was investigated to possess promising anti-inflammatory property through disturbing the activation of NF-κB and ERK/p38/MAPK signaling pathways (Huo et al., 2012). Chen et al. evaluated the anti-inflammatory abilities of alpinetin against LPS-induced mastitis both *in vitro* and *in vivo*. The results displayed that alpinetin evidently weakened neutrophilic granulocytes invasion and the vitalization of myeloperoxidase, decreased TNF-α, IL-1β and IL-6 production by suppressing TLR4/IκB-α/NF-κB signal transduction pathway (Chen et al., 2013). Similarly, He et al. assessed that alpinetin significantly attenuated inflammatory responses in phorbol myristate acetate (PMA)-derived monocytic THP-1 macrophages and dextran sulfate sodium (DSS)-induced acute colitis model. *In vitro*, alpinetin (50–200 μg/ml) considerably decreased the expression levels of TNF-α and IL-1β, and TLR4 mediated NF-κB and NLRP3 inflammasome sensitization. More importantly, administrated with alpinetin (25–100 mg/kg) generated favorable protective effectiveness against diarrhea, colonic shortening and histological injury *in vivo* (He et al., 2016). As reported, alpinetin (at dose of 1 mM) has been explored to improve cartilage degradation and exert excellent anti-inflammatory activities in destabilization of the medial meniscus (DMM)-induced mice osteoarthritis model via blocking the NF-κB/ERK1/2 signaling pathway (Gao et al., 2020). Recently, another report also demonstrated that alpinetin (0.3125–50 mg/ml) effectively suppressed the expression levels of IL-1β, IL-6, inducible nitric oxide synthase (iNOS), TNF-α and human matrix metalloproteinase-13 (MMP-13) gene, and protected the inflammatory damages of chondrocytes induced by LPS *in vitro* (Dai et al., 2020).

Guan et al. has confirmed that oral administrated with alpinetin (10–40 mg/kg) for 7 days exhibited prominently immunomodulatory activity in ConA-induced murine model. The research revealed that alpinetin dramatically restrained murine splenic T lymphocytes growth, CD4^+^ T cell total quantity, Th1/Th2 cytokines secretion, as well as T-cell-mediated delayed-type hypersensitivity reaction tightly correlated with deactivating the immune system aimed NF-kB/NFAT2 pathway (Guan et al., 2014). Thereafter, the researches have described that employing with alpinetin markedly alleviated LPS induced human pulmonary microvascular endothelial (HPMVEC) cells injury *in vitro* and severe acute pancreatitis (SAP)-caused acute lung injury (ALI) *in vivo*. Specifically, alpinetin enabled HPMVEC cells proliferation, enhanced protein aquaporin-1 (AQP-1) generation thereby strengthening water penetrability in the cytomembrane, and restrained pulmonary edema through restricting intercellular cell adhesion molecule-1 (ICAM-1) and TNF-α secretion (Liang et al., 2016; Wang Z. R et al., 2017). In ovalbumin-induced allergic asthma model, Wu et al. illustrated that intraperitoneal administrated with alpinetin (25–100 mg/kg) showed remarkable anti-inflammatory function, and the molecular mechanism was associated with disorganizing PI3K/Akt/NF-κB and activating HO-1 pathways (Wu et al., 2020). Furthermore, Su et al. elucidated that alpinetin was unveiled with a protective effect on chronic obstructive pulmonary disease (COPD). Alpinetin (at dose of 20 mg/kg) treatment positively improved lung function, restored deformation of bronchus lumen, attenuated lumen wall thickness, refrained inflammatory cells infiltration, and inhibited pulmonary fibrosis in alveolar tissue by disturbing inflammatory factors and biochemical markers such as TNF-α, α-smooth muscle actin (α-SMA), IL-6, and transforming growth factor-β1 (TGF-β1), coupled with the downregulation of caspase family (Su et al., 2020).

In summary, these findings demonstrate that alpinetin possesses prospective anti-inflammatory properties through suppressing TLR4/NF-κB, and NF-κB interrelated PI3K/Akt, NFAT2, and ERK/p38/MAPK signaling pathways. Alpinetin is hopeful to be further exploited as a therapeutic agent for instant and chronic inflammatory disorders.

#### Activation of PPAR-γ

Peroxisome proliferators-activated receptor-γ (PPAR-γ), belonging to the nuclear receptor superfamily, is a ligand enabled transcription element which plays critical roles in modulating inflammatory and immune reactions (Vetuschi et al., 2018). Currently, it has been extensively documented that PPAR-γ activators can efficiently block the TLR4/NF-κB signaling pathway and conduct obvious anti-inflammatory activities (Appel et al., 2005).

In THP-1-derived macrophages, Hu et al. found that alpinetin (50–200 μg/ml) markedly excited PPAR-γ and attenuated LPS induced inflammatory mediator response through decreasing TLR4 and NF-κB levels, and inhibiting the phosphorylation of ERK, JNK, and p38 MAPK (Hu et al., 2013). Studies also validated that alpinetin managed curative effects against LPS-induced mice endometritis and carrageenan-induced mice acute inflammation *in vivo*. The results both reported that alpinetin as a PPAR-γ agonist notably suppressed TLR4 signaling pathway and exhibited substantial effect of diminishing inflammation (Liang et al., 2018; Cui et al., 2019). Moreover, Hu et al. calculated that alpinetin administration (50–1,000 μg/ml for 24 h) apparently activated PPAR and reduced proinflammatory cytokines IL-6 generation in RAW246.7 cells. The mechanism analysis clarified that alpinetin firstly enabled PPAR, whereafter activated DNMT3A and boosted cytimidine methylation of the IL-6 promoter region, and finally reduced IL-6 secretion (Hu et al., 2020).

These existed data reveal that alpinetin is a promising agonist for PPAR-γ to remedy inflammatory disorders. Interestingly, PPAR-γ activation could also mediate lipid metabolism, improve insulin sensitivity in adipose tissue, and exert appreciable hypoglycemic function in diabetic patients (Gross and Staels, 2007). Thereby, the hypoglycemic activity of alpinetin is worth anticipating later.

#### Activation of Nrf2/HO-1

Nuclear factor-erythroid 2-related factor 2 (Nrf2) has been ascertained as a well-known redox-sensitive transcriptional regulator of the antioxidants and detoxifying enzymes including glutathione peroxidase, coenzyme II (NADPH) and heme oxygenase (HO-1) (Kubo et al., 2019; Gong et al., 2021). The Nrf2/HO-1 signaling pathway is sensitized in response to ROS, and preserve cells through antagonizing oxidative stress damage triggered by inflammation (Hennig et al., 2018).

For example, treated with alpinetin (25–100 mg/kg) powerfully secured the integrity and perviousness of the intestinal epithelial barrier through regulating tight junction proteins production in DSS-induced ulcerative colitis model. The underlying molecule mechanism was embroiled with enabling the phosphorylation of Nrf2/HO-1 and further restricting oxidative stress (Tan and Zheng, 2018). Identically, a recent study has supported that administrated with alpinetin (40–160 mg/kg) significantly attenuated serious lung damage in cecal ligation and puncture (CLP) provoked sepsis rats through downregulating inflammation and oxidative stress via PI3K/Nrf2/HO-1 pathway (Ren et al., 2021). Hence, it is rational to suppose that Nrf2/HO-1 signaling pathway was involved in the anti-inflammatory activity of alpinetin.

#### Activation of AhR

Aryl hydrocarbon receptor (AhR) is a ligand-respondent transcription factor pertaining to the basic-helix-loop-helix/Per-Arnt-Sim (bHLH/PAS) family (Shinde and McGaha, 2018). AhR has been confirmed to regulate differentiation of multiple T cells such as Th17/Treg balance through binding with both endogenous and exogenous ligands (Neavin et al., 2018).

Lv et al. documented that alpinetin (7.5–30 mg/kg) potently exerted anti-inflammatory effectiveness correlation with AhR activation in DSS-induced mouse colitis. Briefly, alpinetin directly promoted Treg differentiation in CD4^+^T cells of mesenteric lymph nodes (MLNs), restored Th17/Treg balance in colons, and then inhibited symptoms of colitis *in vivo* through elevating AhR expression and coordinating miR-302/DNMT-1/CREB signaling pathway (Lv et al., 2018). Additionally, Miao et al. farther elucidated the mechanism involved in alpinetin regulating mouse colitis. The results demonstrated that alpinetin (3–30 μM) remarkably upregulated AhR level, modified transepithelial electrical resistance in Caco-2 cells induced by TNF-α, decreased the apoptosis and restored the feature of intestinal epithelial cells, and alleviated inflammation *in vivo* by conducting suv39h1, TSC2 and mTORC1 generation (Miao et al., 2019). These data suggest that alpinetin present a unique role in the treatment of ulcerative colitis by vitalizing AhR.

#### Activation of PXR

Pregnane X receptor (PXR), affiliating to the nuclear receptor superfamily, is a transcription factor capable of binding to a broad range of exogenous and endogenous ligands (Mani et al., 2013). Current reports have verified that PXR exerts a key role in eliminating xenobiotic and toxicant, and performing vigorous anti-inflammatory effectiveness against inflammatory bowel disorders (Shah et al., 2007; Cheng et al., 2012).

Recently, Yu et al. investigated that alpinetin bound to PXR-ligand-binding domain as a PXR agonist and notably enabled anti-inflammatory activities in LPS-induced RAW264.7 macrophages, TNF-α-stimulated LS174T colorectal cells, and DSS-induced mice colitis. *In vitro*, administration of alpinetin (25 μM) remarkably inhibited NF-κB activation, and decreased p-p65 production in RAW264.7 and LS174T cells in a PXR dependent manner. Importantly, alpinetin (50 mg/kg) significantly restrained the sensitization of NF-κB, reduced TNF-α, IL-6 and other proinflammatory cytokines expressions, and decreased MPO generation by activating PXR *in vivo* (Yu et al., 2020). The research provided novel perception concerning alpinetin that it manifested as both PXR ligand and activator, and possessed the potential to handle human inflammatory bowel diseases in future.

Additionally, a novel feature of alpinetin was assessed by Liu et al. in ameliorating CLP-induced persistent inflammation, immunosuppression, and catabolism syndrome (PICS). The results exhibited that administration of alpinetin (50 mg/kg intravenously infused for 8 days) remarkably enhanced the survival of septic mice and improved organ dysfunction via inhibiting the release of proinflammatory cytokines, decreasing apoptosis in T lymphocytes, attenuating lung injury, and repressing oxidative stress (Liu et al., 2021). Thereby, alpinetin may be a hopeful curative agentia to prevent PICS. Further clinical trials are inevitable to confirm the therapeutic effects of alpinetin in PICS.

In general, these findings together confirm that alpinetin exerts considerable therapeutic effects on multitudinous inflammatory diseases, including acute lung injury, mastitis, colitis, osteoarthritis, delayed-type hypersensitivity, allergic asthma, COPD, endometritis, and PICS mainly via suppression of NF-κB and excitation of PPAR-γ, Nrf2/HO-1, AhR, and PXR. The energetic anti-inflammatory properties provide convenience for the future clinical utilization of alpinetin.

### Hepatoprotective Activity

Liver is the major organ of human metabolism, and possesses numerous functions including the production of bile, metabolism of nutrients, elimination of endogenous and exogenous substances, glycogen storage, and plasma protein synthesis (Pan et al., 2010; Baghbanan et al., 2014). Recent years, nature flavonoid monomers have emerged as potential hepatoprotective agents due to their safety and efficacy (Ben et al., 2017).

Non-alcoholic fatty liver disease (NAFLD) is reputed as an elementary public health issue globally, which accelerates the pathological progress of various disease such as type II diabetes and cardiovascular disorder (Liu et al., 2014; Sheldon et al., 2014). Zhou et al. certified that alpinetin (at dose of 12.5–50 mg/kg) obviously ameliorated high fat diet-induced NAFLD in mice. The results revealed that alpinetin considerably suppressed oxidative stress and inflammatory damage through promoting PPARα/SOD1/HO-1/Nrf2 secretion and inhibiting the activation of TLR4/NF-κB pathway. Moreover, alpinetin strongly moderated abnormal lipids metabolism in NAFLD by decreasing thioredoxin-interacting protein (TXNIP)/xanthine oxidase (XO), Stearoyl-CoA desaturase1 (SCD1), and fatty acid synthase (FAS) expression levels (Zhou et al., 2018). Liu et al. assessed the therapeutic effect of alpinetin in LPS/D-Gal (Lipopolysaccharide/d-galactosamine)-induced mice liver damage model. Alpinetin administration (12.5–50 mg/kg) appreciably attenuated the activation of NF-κB, enhanced Nrf2 generation, and ultimately decreased inflammatory and oxidative response *in vivo* (Liu et al., 2019). Whereafter, Pan et al. illustrated that alpinetin exhibited profitable protective effects on hepatic ischemia/reperfusion (I/R) injury both *in vitro* and *in vivo*. The study demonstrated that alpinetin (50 mg/kg) suppressed the expression of alanine aminotransferase, aspartate transaminase and proinflammatory cytokines, and inhibited the pathological progress of hepatocyte damage caused by hepatic I/R via inhibiting NF-κB/MAPK signaling pathways (Pan et al., 2021). Interesting, alpinetin also exerted anti-fibrotic effect in mice model with carbon tetrachloride (CCl_4_)-induced liver fibrogenesis. Alpinetin treatment (15 and 60 mg/kg) displayed anti-inflammatory and anti-oxidative properties through reducing NLRP3 expression level, activating Nrf2 pathway, and limiting hepatic angiogenesis (Zhu et al., 2021).

As mentioned above, alpinetin is capable of exhibiting potential therapeutic effects in the treatment of various hepatic disorders by facilitating the activation of PPARα, SOD1, HO-1 and Nrf2, dropping the expression of NLRP3, TXNIP, XO, SCD1, FAS, and inhibiting TLR4/NF-κB and MAPK signaling pathways (Table 2). However, many challenges for researchers are still remained. More specific and novel molecular targets of alpinetin in hepatic disorders therapy are required to be illustrated in further experiments, such as glucagon-like peptide-1 (GLP-1) (Milani et al., 2019), Sirtuin 1 (SIRT1) (Farghali et al., 2019), Yes-associated protein (YAP) (Xie et al., 2021), etc.

### Cardiovascular Protective Activity

Cardiovascular disease (CVD) has become the largest cause of morbidity and premature death worldwide. According to some estimates, the number of CVD-attack deaths in the world exceed 17 million per year (Haouari and Rosado, 2019; Aryan et al., 2020). The most commonly cardiovascular risk factors are age, gender, genetic factors, atherosclerosis, cardiac failure, obesity, coronary heart disease, hypertension, hyperglycemia and dyslipidemia (Ciumărnean et al., 2020). As a promising reagent for cardiovascular disorders, alpinetin exhibited multiple therapeutic effects targeted on platelets, myocardial cells, vascular smooth muscle cells, and lipid accumulation (Table 2).

#### Protective Effect on Atherosclerosis

Atherosclerosis is one of the primary causes contributed to cardiovascular disease. Atherosclerosis is distinguished by the lipids abnormally accumulated in the arteriosus wall (Hansson and Hermansson, 2011). Simultaneously, platelet is marked as the critical factor in thrombus and atherosclerosis during the pathogenetic process (McNicol and Israels, 2003; Kang et al., 2013). In the study evaluated by Jantan et al., alpinetin represented conspicuous inhibitory effects on platelet-activating factor (PAF) with IC_50_ values of 41.6 μM, suggesting that alpinetin was relatively potent PAF receptor binding inhibitors (Jantan et al., 2004). Enabling cholesterol exocytosis from lipid-loaded cells is a reliable strategy for the treatment of atherosclerosis (Luo et al., 2010). Jiang et al. elucidated that administrated with alpinetin (50–150 μg/ml for 24 h) exhibited profitable effect on cholesterol transportation in human peripheral blood monocyte derived macrophages (HMDMs) and THP-1 macrophage cells. The data obtained demonstrated that alpinetin significantly strengthened cholesterol excretion, restricted oxidized low-density lipoprotein (ox-LDL)-induced lipid aggregation by enhancing PPAR-γ, LXRα, ABCA1 and ABCG1 levels (Jiang et al., 2015). Vascular smooth muscle cells (VSMC) are a major cell type present at the pathology of occluding arterial lesions during atherogenesis (Grootaert et al., 2018). Li et al. investigated that alpinetin appreciably refrained VSMC growth and invasion, and secured VSMC against peroxide injury activated by TNF-α and H_2_O_2_ through decreasing NO and LDH expressions (Li and Du, 2004). Besides, atherosclerosis is compactly connected with coronary heart disease (CHD), which is featured with the presence of arterial plaques principally constructed by fatty substance, calcium and inflammatory cells (Li et al., 2018). Dai et al. recently reported that alpinetin (40–160 mg/kg) notably attenuated left ventricular end-diastolic volume (LVEDV), inhibited serum triglyceride (TG), endothelin-1 (ET-1) and TNF-α production, upregulated NO expression, and finally improved heart function in CHD rat model through suppressing MEK/ERK signaling pathway (Dai et al., 2021).

Based on these findings, we are able to summarize that alpinetin may hold a bright future to treat atherosclerosis and its complications by restraining PAF, promoting cholesterol efflux, protecting VSMC, and ameliorating cardiac functions.

#### Inhibition of Myocardial Apoptosis

Myocardial apoptosis, predominantly induced in myocardial ischemia, anoxia and ischemia-reperfusion (Gottlieb et al., 1994), is an important cytological factor in the evolution of numerous heart diseases. Suo et al. found that alpinetin administration (at dose of 40–120 mg/ml) demonstrated satisfactory therapeutic effect on myocardial cell apoptosis activated by serum expropriation in rats. The research revealed that alpinetin stimulated the δ receptor, thus sensitized PKC/ERK signaling pathway and inhibited caspase family generation, and eventually improved the intrinsic protection in myocardial cells (Suo et al., 2014).

#### Relaxation of Vascular

Vasodilators are efficient tactics to remedy diverse cardiovascular diseases, including hypertension, acute heart failure and cardiac arrythmias (Allam et al., 2020). Previous research substantiated that alpinetin (10–100 μM) evidently exerted vascular relaxation functions in both endothelium-dependent and independent manner. The results suggested that alpinetin relaxed rat mesenteric arteries through activating nitric oxide expression, unconditionally restraining Ca2^+^ influx, limiting intracellular Ca2^+^ release, and inhibiting protein kinase C regulated excitation-contraction coupling (Wang et al., 2001).

Therefore, similar to many drugs clinically applicated in patients with cardiovascular diseases, alpinetin enjoys multiple directions and targets for CVD treatment, including anti-atherosclerosis, curbing myocardial apoptosis, and loosing vascular. Although alpinetin seems to hold the capacious therapeutic prospect for CVD, further in-depth researches need to be pursued.

### Antimicrobial Activity

In recent years, the anti-infective research of natural flavonoids has attracted extensive attention. Numerous research teams have isolated and identified the structures of flavonoids, and investigated the antibacterial and antiviral activities of these compounds (Cushnie and Lamb, 2005). Foregoing studies reported that alpinetin displayed broad spectrum antibacterial activity, particularly against *Helicobacter pylor*i (Huang et al., 2006). The bioactive minimum inhibitory concentration (MIC) values of alpinetin against *Helicobacter pylor*i was 1.25 μg/ml. Moreover, alpinetin was active against Gram-negative bacteria such as *Escherichia coli*, *Salmonella typhi*, *Klebsiella penumoniae*, *Pseudomonas pyocyanea*, *Enterobacter aerogenes*, *Pseudomonas maltophilia*, *Citrobacter diversus*, and *Pseudomonas cepacian* with the MIC ranged from 1.925 to 3.859 mg/ml (Huang et al., 2006). Besides, Chen et al. presented that alpinetin performed excellent antibacterial activities in drug-resistant *Aeromonas* hydrophila (CW, 1G, Ah, WZ1 and S1D) *in vitro*. The results found that alpinetin significantly refrained the proliferation of five fish-differentiated *Aeromonas* hydrophila coupled with the MIC and MBC (minimum bactericidal concentration) ranged from 128–256 μg/ml to 512–1,024 μg/ml, respectively. Afterwards, the hidden mechanism was testified, and the results indicated that the antibacterial profile of alpinetin was prominently carried out through destructing cell walls of bacteria as well as promoting the penetrability of cell membranes (Chen H et al., 2021).

To sum up, alpinetin may possess a wide antimicrobial spectrum and robust antimicrobial activity (Table 2), and can be used for further antimicrobial drug discovery. However, there are few reports focused on this domain, nor systemic researches on the antibacterial mechanisms of alpinetin, which will be the hits of future studies.

### Antiviral Activity

Respiratory syncytial virus (RSV), parainfluenza type 3 (Para 3), and influenza type A (Flu A) are often the cause of severe respiratory diseases (Freymuth et al., 2004). Alpinetin was explored for inhibitory effects against these kinds of viruses utilizing cytopathic effect analysis in cell culture monomolecular layers. Importantly, alpinetin exhibited conducive antiviral activity against RSV (IC_50_ = 77.0 μM), Para 3 (IC_50_ = 154.4 μM), and Flu A (IC_50_ = 308.5 μM) with a therapeutic index (TC_50_/IC_50_) surveyed to 6.0, 3.0 and 1.5, respectively (But et al., 2009). Moreover, the HIV-1 pandemic is undoubtedly the defining world-wide health crisis (Simon et al., 2006). Alpinetin has been anteriorly separated from the ethanol extract of *Boesenbergia rotunda* (L.) Mansf. [Zingiberaceae] in Thailand, and was reported to exert inhibitory activity against HIV-1 protease (HIV-PR), which is identified as a crucial molecular marker for promoting targeted drugs against HIV (Tewtrakul et al., 2003; Maridass et al., 2008). Thereafter, Pan et al. isolated alpinetin from the branches and leaves of *Vitex tripinnata* (Lour.) Merr. [Lamiaceae] using bioassay-guided fractionation, and the antiviral activities of alpinetin has also been investigated. The results suggested that alpinetin possessed anti-HIV property with IC_50_ values of 130 μM (Pan et al., 2014). Recently, severe acute respiratory syndrome coronavirus 2 (SARS-CoV-2), an extremely contagious RNA virus, is liable for contributing to the coronavirus disease 19 (COVID-19) pandemic (Hu et al., 2021). Gurung et al. applied methods of molecular docking and dynamic simulation to assess that alpinetin could considerably block the reproduction of SARS-CoV-2 by targeting the main protease. More specifically, alpinetin strongly bound to the active site pocket of SARS-CoV-2-Main protease principally stimulated by van der Waals forces. The binding energy and inhibition constant of alpinetin were -7.51 kcal/mol, and 3.12 μM, respectively (Gurung et al., 2021). Therefore, alpinetin may be further developed as a promising anti-SARS-CoV-2 candidate.

These researches uncover that alpinetin may hold inhibitory effects against diverse viruses (Table 2). But the potential antiviral mechanisms majorly remain unclear and need to be probed. More interestingly, it will also be deserved to explore the pharmacological roles of alpinetin in the confrontation of new emerging strains.

### Other Pharmacological Properties

AChE, a member of the hydrolase enzyme family, has cholinergic roles in the breakdown of acetylcholine (ACh) neurotransmitters and terminating cholinergic signaling in mammals (Akıncıoğlu and Gülçin, 2020). Alpinetin has formerly been certified to activate AChE expression by modulating G protein-coupled receptor 30 (GPR 30) in PC12 cells *in vitro* (Liu et al., 2019) (Table 2). Despite there are few analogical studies of alpinetin in neurology, Liu et al. provided a beneficial preliminary focused on nervous disorder. Therefore, future researches should be carried out to determine if alpinetin can be developed as a promising candidate for some possible brain diseases.

Flavonoids are universally known as efficient antioxidants account for the aspect of endowing phenolic hydrogens (Pietta, 2000). Recently, studies substantiated that several antioxidative flavonoids could promote photo protection and perform as UV filters to avert DNA injury, skin canceration, sun burn, etc (González et al., 2008). Shireen et al. assessed the antioxidative activity and UV spectral features of alpinetin by computational investigations, Time dependent density functional theory (TD-DFT) and Natural bond orbital (NBO) methods. The results revealed that alpinetin possessed a potential antioxidative effect, and displayed an extensive absorption in the extent of harmful UV radiation (270–390 nm) due to the crucial configurable feature with the absence of C2-C3 double bond (Shireen et al., 2017) (Table 2). Therefore, alpinetin can be regarding as a hopeful candidate in future research for antioxidative UV filters in sunscreens.

Additionally, exploring the interactions between flavonoids with protein or DNA can afford valuable information of the structural features and pharmacodynamics of potential compounds (Pal and Saha, 2014; Selvaraj and Singh, 2018). For example, alpinetin bound on site III and led to a conformational change of human serum albumin (HSA). The binding features and conjugation site positions are potential to prohibit some harmful drug reactions such as hypoglycemia (He et al., 2005). Thereafter, He et al. also studied the interactions between alpinetin and lysozyme. The results demonstrated that alpinetin exerted excellent affinity to lysozyme spurred by hydrophobic and electrostatic effects with the binding distance of 4.04 nm. The binding function provided avenues to ascertain the toxicity effects of alpinetin on target proteins (He et al., 2006). Similarly, alpinetin could bind to the subdomain IIA of Bovine serum albumin (BSA) through hydrophobic force without non-radioactive energy transfer, and the binding effect induced some microenvironmental and conformational change of BSA (Ni et al., 2010; Zhang et al., 2010). Moreover, Xu et al. investigated the underlying mechanism of alpinetin interaction with bovine hemoglobin (BHG) under physiological conditions. The data elucidated that alpinetin strongly bound to the hydrophobic lacuna of BHG driven by hydrophobic effect, and extinguished the immanent fluorescence of BHG related with altering conformation, hinting that alpinetin could be reserved and delivered by BHG to some extent (Xu et al., 2017). On the other side, Zhang et al. studied the recognition between alpinetin and calf thymus DNA. Fluorescence and UV-visible spectrometry experiments demonstrated that alpinetin bound to DNA with a groove and single static model (Zhang et al., 2008). Furthermore, recent reports found that alpinetin bound to the cavity of R273H mutant p53 through mediately or directly impacting the DNA binding domain with a dissociation constant calculated to 75.11 µM, indicating that alpinetin may definitely salvage DNA-contact mutant p53 in tumor treatment (Malami et al., 2017b). Summarizing these binding effects of alpinetin, we may able to deduce that alpinetin is a capable and valid candidate binding on DNA or protein to prevent and treat diseases.

Besides, Lu et al. investigated the inhibition activities of alpinetin on seven major cytochrome P450 monooxygenases (P450s), including CYP1A2, CYP3A4, CYP2E1, CYP2D6, CYP2A6, CYP2C8, and CYP2C9 in human liver microsomes. Alpinetin only competitively inhibited CYP1A2 at the concentration of 100 μM, indicating that it may be used as a selective CYP1A2 inhibitor (Lu et al., 2015). Furthermore, alpinetin notably transactivated the CYP3A4 in LS174T cells with 10 μM via activation of the PXR pathway (Dou et al., 2012). These reports implied that alpinetin may be a potential regulator in the metabolisms of CYP1A2 and CYP3A4 mediated drugs.

## Pharmacokinetics and Enhancement Strategies

### Pharmacokinetics

Pharmacokinetics is mainly to quantitatively estimate the absorption, distribution, metabolism and excretion properties of drugs, which provide essential information for clinical research (Rock and Foti, 2019; Wang et al., 2021). Recently, the pharmacokinetic profiles of alpinetin have been investigated by ultrahigh performance liquid chromatography-tandem mass spectrometry (UHPLC-MS/MS) (Ye et al., 2018), ultra-performance liquid chromatography tandem mass spectrometry method with electrospray ionisation (UHPLC-ESI-MS/MS) (Chen et al., 2015), and ultra-performance liquid chromatography quadrupole time-of-flight mass spectrometry (UPLC-Q-TOF-MS) primary in rats (Qiu et al., 2019) (Table 3).

**TABLE 3:** The pharmacokinetics of alpinetin.

Administration	Species	Doses	Pharmacokinetic parameters/Detail							References
			T_1/2_ (h)	C_max_ (μg/L)	AUC_(0-t)_ (μg/L×h)	MRT_(0-t)_ (h)	T_max_ (h)	CL (L/h/kg)	V (L/kg)	
i.g.	Rat	5 mg/kg	1.578 ± 0.239	385.633 ± 91.192	911.723 ± 59.208	1.997 ± 0.069	-	10.683 ± 0.684	24.295 ± 6.858	Chen et al. (2015)
i.g.	Rat	20 mg/kg	9.049 ± 4.21	167.020 ± 43.958	783.623 ± 296.957	7.676 ± 0.375	0.125 ± 0.083	26.327 ± 13.708	337.314 ± 233.771	Ye et al. (2018)
i.v.	Rat	2 mg/kg	7.768 ± 4.695	686.471 ± 73.139	518.945 ± 159.366	1.941 ± 0.400	-	3.993 ± 1.189	50.689 ± 42.720	Ye et al. (2018)
i.g.	Rat	40 mg/kg	Alpinetin was undergone significant glucuronidation in rats, all together 15 metabolites of alpinetin were detected in plasma, urine, bile and feces							Qiu et al. (2019)

Reports documented that alpinetin was rapidly absorbed into- emocircular system. Specifically, the elimination half-life (T_1/2_) after oral administrated with alpinetin (5 and 20 mg/kg) were 1.578 ± 0.24 h and 9.049 ± 4.21 h, respectively. Meanwhile, area under the curve (AUC_0∼t_), maximum concentration (C_max_) and total clearance (CL) were 783.623 ± 296.957 μg/L×h, 385.633 ± 91.192 μg/L and 10.683 ±0.684 L/h/kg, and 906.058 ± 402.669 μg/L×h, 167.020 ± 43.958 μg/L and 26.327 ± 13.708 L/h/kg, respectively (Chen et al., 2015; Ye et al., 2018). Although the results varied probably attributed to the application of different dosages and detection methods. The conjectures could be drawn that alpinetin might present pharmacological activity rapidly and subject the elimination process quickly. Comparing with iv dosing (2 mg/kg), the absolute bioavailability of alpinetin was 15.10 ± 5.72%, suggesting that alpinetin has a poor intestinal absorption (Ye et al., 2018). Thereby, these pharmacokinetic studies displayed a fast absorption, poor bioavailability, and rapid clearance *in vivo*, which will severely hinder its therapeutic effectiveness.

Glucuronidation, a typical phase II metabolic reaction catalyzed by UDP-glucuronosyltransferases (UGTs), is identified as the prominent metabolic pathway for flavonoid monomers *in vivo* (Tang et al., 2009). Qi et al. elucidated the glucuronidation metabolic characteristics of alpinetin enabled by intestine and human liver microsomes *in vitro*. The research indicated that enzymes from UGT1A1, UGT1A3, UGT1A9, and UGT2B15 subfamilies are the main contributors to accelerate alpinetin metabolism. Importantly, in addition to UGTs, breast cancer resistance protein (BCRP) has been confirmed as another critical factor in regulating the dramatical glucuronidation of alpinetin. The suppression of UGTs and BCRP both decreased cellular glucuronidation and may be helpful to improve pharmacokinetic features of alpinetin *in vivo* (Qi et al., 2019). Afterwards, Qiu et al. verified that alpinetin experienced notable glucuronidation in rats. The results validated that alpinetin primarily absorbed into the small intestine with the manners of phenolic acids and prototype. The entered substrates were markedly glucuronidated to form glucuronide conjugates in the blood and liver, then converted into bile or blood circulation, and finally excreted through urine and feces (Qiu et al., 2019).

### Enhancement Strategies

Since the terrible pharmacokinetics profiles may exceeding dampen the *in vivo* bioactivity of alpinetin, more attention should be focusing on some feasible means to improve its activity in ameliorating various human ailments.

Hydroxypropyl-β-cyclodextrin host-guest system is a useful approach to satisfactorily improve drug solubility, stability as well as bioavailability (Garnero et al., 2012). Ma et al. firstly demonstrated that alpinetin obviously enhanced the water solubility and stability in the inclusion complex with HPβCD, which may be beneficial for alpinetin in clinical application (Ma et al., 2012). The inclusion complexation was a critical step for alpinetin to devise novel dosage forms. Additionally, other encapsulation systems, such as microemulsion and phospholipid entrapment, have been designed to effectively deliver flavonoids inside the body (Vazhappilly et al., 2021). The possibility of utilizing these systems may improve the bioavailability and curative effects of alpinetin.

Recently, the rapid nanoscience development has harvested affluent admiration in the pharmaceutical industry. Novel nanotechnologies mainly included nanoparticles, nanogel, nanocrystals, nanoemulsion, nanosuspension, micelles, liposome, solid lipid nanoparticles, self-nanoemulsifying drug delivery systems (SNEDDS), and self-micro-emulsifying drug delivery system (SMDDS) (Ahmad et al., 2016; Alothaid et al., 2021; Jain et al., 2021). Utilizing nanotechnology has created foremost progresses toward enhancing stability, bioavailability, delivery, sustained release, and therapeutic index of flavonoids (Ayala-Fuentes and Chavez-Santoscoy, 2021; Jannat et al., 2021). Considering the promising therapeutic effects of alpinetin, these novel nano-drug delivery systems as prospective approaches should be exploited to deliver alpinetin in a managed and specific way.

Making a comparison with flavonoid leads, methylated flavonoids have been elucidated to possess much better bioavailability (Wen et al., 2017). The structure-activity relationships of methylated alpinetin may help to overcome the following challenges, either in poor bioactivity or in alpinetin-based drug discovery. Furthermore, given the significant glucuronidation of alpinetin, it may be a potent approach to upgrade the bioavailability by combining relevant UGTs inhibitors, such as piperine and quercetin (Zeng et al., 2017).

## Conclusion and Future Perspectives

Alpinetin is a nature flavonoid present in abundant medicinal plants. Up to now, alpinetin is reportedly documented against various diseases encompassing malignancies, inflammation, liver disorders, cardiovascular diseases, bacterial infections, virus infections, lung injury, brain disease, and oxidative damage. The reported antineoplastic aspects are mainly associated with ROS/NF-κB/HIF-1α, PI3K/Akt/mTOR, STAT3/c-Myc/survivin, UCK2/MDM2/p53, PPAR-γ, Notch, and MKK7/JNK pathways. Alpinetin exerts noteworthy anti-inflammatory functions via modulating PI3K/Akt, TLR4/NF-κB, ERK/JNK/p38 MAPK, PPAR-γ, PXR, Nrf2/HO-1, AhR/miR-302/DNMT-1/CREB, and AhR/suv39h1/TSC2/mTORC1 signaling pathways in versatile inflammatory models. The excellent anti-inflammatory, anti-oxidation and lipid metabolism amelioration properties both devote to notable hepatoprotective activities of alpinetin, making it a prospective reagent for remedying diverse hepatica illness, including liver injury, NAFLD, and liver fibrosis. Many studies reveal that alpinetin performs profound therapeutic effects on various cardiovascular diseases through anti-platelet, anti-atherosclerosis, anti-coronary heart disease, anti-myocardial apoptosis, and vasodilation. Alpinetin shows expansive antibacterial property especially against *Helicobacter pylori*, mainly involved in demolishing cell walls and enhancing the penetrability of cell membranes. Alpinetin also possesses considerable antiviral activities against RSV, Para 3, Flu A, HIV, and SARS-CoV-2. The antioxidative activity and UV spectral features endow alpinetin with the capacity to reduce UV-induced skin damage. Moreover, alpinetin holds powerful binding effects toward versatile DNA and proteins. All current evidence demonstrates that alpinetin can be application in the development of drugs that could be used in various diseases managements. However, more pharmacological researches are needed to elucidate novel viewpoints for disease treatment in the field of modern medicine.

Besides medicinal plants, the present vegetables and fruits extensively used as daily food are also known to possess a high concentration of flavonoids. Flavonoids are strong nutraceutical and medicinal candidates due to their pharmacological activities and safety. Alpinetin has been frequently applied in compound preparations, and mainly confirmed to include slight systemic toxicity. However, reports in this crucial filed are deficient and limited. It should be point out that sufficient toxicological researches are imminent to justify clinical safety of alpinetin. Drug development usually meets huge hurdles such as poor pharmacokinetic properties and crappy *in vivo* activity. Despite the current pharmacokinetic studies are not adequate, the facts they revealed can not be neglected. Focused endeavors are needed to improve the bioactivity of alpinetin through viable strategies as we aforementioned or others, such as encapsulation systems, nano-drug delivery systems, chemical modification, adjuvants combination, etc. Therefore, for development of alpinetin finally into successful drug, future researches need to concentrate upon the evaluation of complete toxicological effects and the enhancement of pharmacokinetic profiles with scientific and technological advances.

## Glossary

PARP: poly ADP-ribose polymerase
Bcl-2: B-cell lymphoma-2
Bax: BCL2-Associated X
NF-κB: nuclear factor kappa-B
IκBα: inhibitor of NF-κBα
HIF-1α: hypoxia-inducible factor-1α
MuRF1: muscle RING-finger protein-1
Atrogin-1: muscle atrophy gene‐1
CDK: cyclin-dependent kinases
FASN: fatty acid synthase
SCD: stearoyl CoA desaturase
F4/80: mouse EGF-like module-containing mucin-like hormone receptor-like 1
IL: interleukin
CCL: chemokine (C-C motif) ligand
XIAP: X-linked inhibitor of apoptosis protein
Akt: protein kinase B
PI3K: phosphoinositol-3 kinase
MRP: multidrug resistance-associated protein
P-gp: P-glycoprotein
TIMP: tissue inhibitor of metalloproteinase
MMP: matrix metalloproteinase
STAT3: signal transducer and activator of transcription 3
NICD: notch intracellular domain
HES: hairy and enhancer of split
MKK-7: Mitogen-activated protein kinase kinase-7
UCK2: uridine-cytidine kinase 2
MDM2: human double minute 2
TNF-α: tumor necrosis factor-α
LPS: lipopolysaccharide
ERK: extracellular signal-regulated kinase
PPAR: peroxisome proliferator-activated receptor
MPO: myeloperoxidase
TLR4: Toll-like Receptor 4
DSS: dextran sulfate sodium
NLRP3NOD-: NOD-LRR- and pyrin domain-containing protein 3
ASC: apoptosis-associated speck-like protein containing a CARD
JNK: c-Jun N-terminal kinase
DMM: destabilization of the medial meniscus
COL2A1: collagen type II
ADAMTS: a disintegrin and metalloprotease with thrombospondin type 1 motifs
iNOS: inducible nitric oxide synthase
AQP-1: protein aquaporin
ICAM-1: intercellular adhesion molecule-1
IFN-γ: interferon-gamma
MIP-2: macrophage inhibitory protein-2
SOD: dismutase
MDA: melanoma differentiation associate
GSH: glutathione
Nrf2: nuclear factor-erythroid 2-related factor 2
HO-1: heme oxygenase-1
RORγt: retinoic acid-related orphan receptor-γt
FOXP3: ranscription factor forkhead box protein P3
AhR: aryl hydrocarbon receptor
ARNT: AhR-nuclear translocator
CREB: cAMP-response element binding protein
ZO-1: zonula occluden-1
LC3B: microtubule-associated protein light chain 3B
Atg: autophagy-related proteins
p70S6K: 70 kDa ribosomal S6 kinase
TSC: tuberous sclerosis complex
PTEN: p and tensin homolog
AMPKα: AMP-activated protein kinase alpha
TGF-β1: transforming growth factor-β1
α-SMA: α-smooth muscle actin
ALT: alanine aminotransferase
AST: aspartate aminotransferase
MCP-1: monocyte chemoattractant protein-1
IKKβ: nuclear factor kappa B kinase subunit beta
LDH: lactate dehydrogenase
CAT: catalase
GPx: glutathione peroxidase
VEGF: vascular endothelial growth factor
PDGF: platelet-derived growth factor
GCLC: glutamate-cysteine ligase catalytic subunit
NQO1: NAD(P)H quinone dehydrogenase 1
GCLM: glutamate-cysteine ligase modifier subunit
TXNIP: thioredoxin-interacting protein
SREBP-1c: sterol element regulatory binding protein 1c
LXR-α: Liver X Receptor-α
Elovl-2: elongases of very long-chain fatty acids-2
IRS1: p-insulin receptor substrate 1
ABCA1: ATP binding cassette transporter A1
ABCG1: ATP binding cassette transporter G1
PKC: Protein kinase C
NO: nitric oxide
AChE: acetylcholinesterase
NFs: neurofilaments
MIC: minimum inhibitory concentrations
IC50: half maximal inhibitory concentration
MBC: minimum bactericidal concentration
LVEDV: Left ventricular end-diastolic volume
SV: stroke volume
TG: triglyceride
TC: total cholesterol
HDL-C: high density lipoprotein cholesterol
LDL-C: Low-density lipoprotein cholesterol
ET-1: Endothelin-1
PGI2: prostacyclin
PXR: Pregnane X receptor
i.v.: intravenous administration
i.p.: intraperitoneal administration
i.g.: intragastric administration
T1/2: elimination half-life
AUC0∼t: area under the curve
Cmax: maximum concentration
CL: total clearance
V: apparent volume of distribution
MRT(0-t): mean residence time
Tmax: peak time
